# The impact of feature combinations on machine learning models for in-hospital mortality prediction

**DOI:** 10.1038/s41598-025-26611-y

**Published:** 2025-11-07

**Authors:** Eline Stenwig, Pierluigi Salvo Rossi, Giampiero Salvi, Nils Kristian Skjærvold

**Affiliations:** 1 Department of Circulation and Medical Imaging, The Norwegian University of Science and Technology, Trondheim, Norway; 2 Department of Electronic Systems, The Norwegian University of Science and Technology, Trondheim, Norway; 3 Clinic of Anaesthesia and Intensive Care Medicine, St. Olav’s University Hospital, Trondheim, Norway

**Keywords:** Machine learning, Explainability, XAI, Mortality prediction, XGBoost, Feature selection, EICU, SHAP values, Health care, Mathematics and computing, Computational biology and bioinformatics

## Abstract

The growing volume of healthcare data presents opportunities for machine learning to improve treatment, uncover new patterns in data and predict patient outcomes. Selecting appropriate features for a machine learning model is an important step in the process as the choice of relevant variables directly influences the model’s performance and interpretability. Effective feature selection can enhance both the accuracy and generalisability of the model, especially given the complexity and heterogeneity of healthcare data. The XGBoost algorithm is trained on the eICU Collaborative Research Database to predict in-hospital mortality, with focus on investigating the impact of different feature sets. The analysis cohort comprised 73 210 patients. Different models are trained and tested using 20 000 distinct feature sets, each containing ten features, to assess how different features influence model performance. The models are trained using a train/test split of 80/20. Shapley additive explanations (SHAP) values are used to evaluate the importance of individual features. On average, the feature sets achieve an area under the receiver operating characteristic curve (AUROC) of 0.811, with the highest AUROC of 0.832 obtained from the feature set comprising [admission diagnosis, age, albumin, creatinine, heart rate, mean blood pressure, motor (from Glasgow Coma Scale), respiratory rate, temperature, unit admit source]. Despite variations in feature composition, models exhibit comparable performance in terms of both AUROC and the area under the precision-recall curve (AUPRC). Overall, *age* emerges as particularly influential, appearing most frequently in the feature sets associated with the highest AUROC scores. However, this trend is not observed for AUPRC. The results show that different models can achieve similar discrimination for different feature sets and that feature importance and ranking vary accordingly. This suggests that there may be multiple routes to good performance and that evaluating several feature combinations could be more informative than focusing on a single best set. Average feature importances may not reliably indicate a variable’s overall utility or real-world importance and should be interpreted within the context of specific combinations. Prospective evaluation of promising sets and attention to robustness across combinations may help guide validation and eventual clinical use.

## Introduction

The volume of data generated in healthcare is increasing rapidly, driving interest in leveraging this information with the help from machine learning (ML) models in order to improve treatment, uncover new data patterns and predict patient outcomes^1^. However, developing such models requires careful planning and execution. Healthcare data is highly heterogeneous, with significant variation in quality. Missing or incomplete data is common, and the selection of measurements available may differ considerably between patients depending on where they are admitted, what type of symptoms and diagnosis they have, and the tests offered by the hospital. These factors, and many more, complicate the use and transferability of ML models in healthcare.

An essential step in ML development is determining which features or variables to include in the model. Models with fewer features are typically simpler, faster, and less prone to overfitting. Lower complexity may increase model interpretability but can also compromise performance due to insufficient information. Models with a greater number of features can capture more information but typically require larger sample sizes to generalise to unseen samples^2^. When the data collection requires manual processes or multiple sources, limiting the number of features simplifies this work. One approach to discovering an optimal feature set is to test every possible combination of features and select the best one. However, this strategy is only feasible for datasets with small feature spaces, as the number of feature combinations increases exponentially with the number of features.

This study looks at the binary classification task of in-hospital mortality prediction in the intensive care unit (ICU) as a case study to explore different feature sets. Previous research on mortality prediction models utilises different methods for determining which features to use. Some studies base the selected features either fully or partially on medical domain knowledge of experts and/or literature^3–5^, or on one or more established scoring systems^4,6,7^. Supervised feature selection methods are more common than unsupervised ones and are used for both univariate and multivariate feature selection^5,8,9^. Other studies add or remove variables based on dataset properties such as missing values^4^, or other criteria such as using only non-invasive parameters^10^. Testing multiple feature sets to determine the best one is also possible^8^.

Recent literature highlights the importance of understanding how different features contribute to model predictions^11^, serving as a tool for both feature selection^12,13^ and for enhancing model explainability^14^.

In our previous work^15^, we showed that models with similar performance in terms of AUROC could produce very different interpretations of feature importance when using the same feature set, both at the population level and for individual predictions. This highlighted how model interpretability can vary significantly despite comparable discriminatory performance. The current study explores a different but related question. Instead of focusing on interpretability within a fixed feature set, this study aims to investigate issues and provide further insights to the reliability of ML models based on different feature sets. Specifically, we explore how models behave and how they interpret features when the feature combination changes. By comparing performance and feature importance of different ML models, we investigate how features and feature set combinations impact the models. The aim is not to develop a better model or method but to bring attention to some of the challenges and considerations that arise when selecting and interpreting features across varying feature sets.

## Methods

### Dataset

The dataset used is extracted from the publicly available eICU Collaborative Research Database^16^. The database includes over 200 000 ICU stays from over 130 000 patients in different hospitals in the US between 2014 and 2015. The database comprises multiple tables containing information about demography, vital and lab values, and care provided. The database also includes dedicated tables for the severity-of-illness score APACHE IV, along with the variables used to calculate it.

### Patient selection

**Fig. 1:**
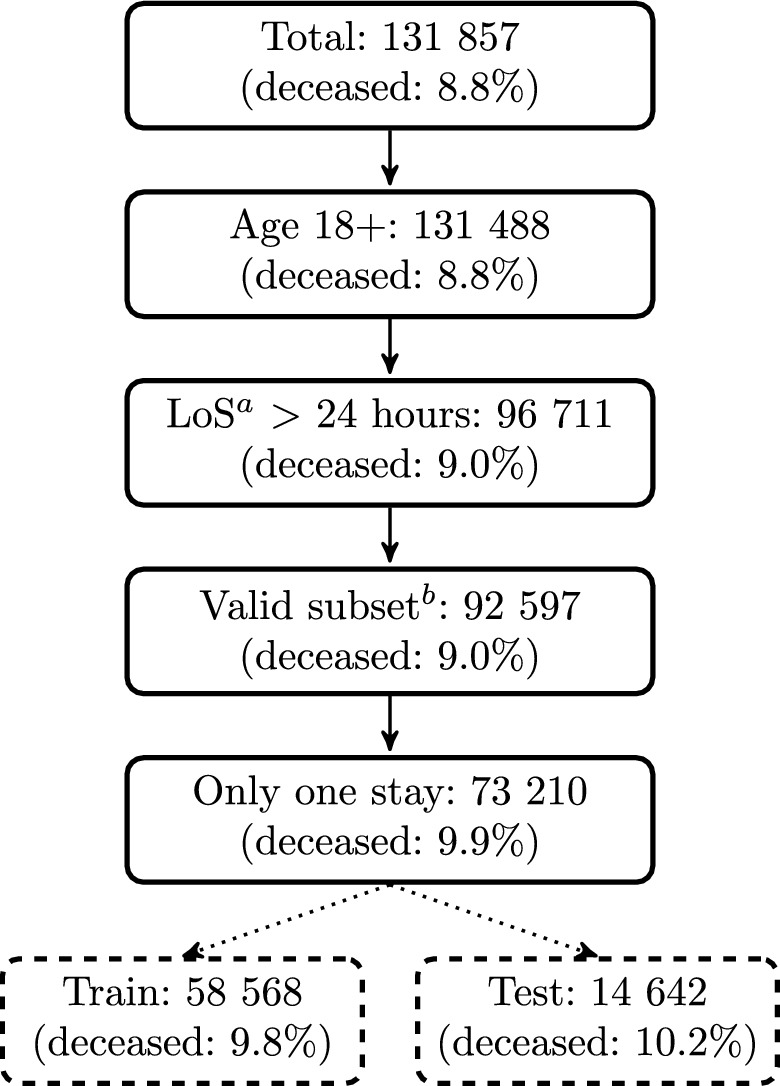
Patient selection. Patient selection criteria and train/test splitting of dataset. Superscript a: length of stay. Superscript b: patients with missing or unknown *age*, *gender*, patient id, hospital discharge status, *admissionheight*, *admissionweight*, and predicted hospital mortality. Outliers for *admissionheight* and *admissionweight* are removed.

The patient selection process is shown in Fig. 1. Patients with multiple stays are excluded to avoid repeated measurements, as are patients below 18 years to include solely the adult population. Patients with a length of stay shorter than 24 hours are omitted as the feature values for the APACHE IV score are computed from the worst value within the first 24 hours of the ICU stay. Patients with missing or unknown *age*, sex, patient id, hospital discharge status, admission height, admission weight, and APACHE IV predicted hospital mortality are also excluded as well as patients with unrealistic outliers for height and weight.

### Machine learning algorithm

The models are developed using the eXtreme Gradient Boosting (XGBoost) algorithm. XGBoost combines multiple weak decision tree models to form a strong one. The algorithm handles missing values by default, eliminating the need for manual value imputation. The XGBoost algorithm is fast, computationally inexpensive, and has achieved good performance on numerous machine learning tasks^17–19^.

### Performance evaluation

The area under the receiver operating characteristics curve (AUROC) and the area under precision-recall curve (AUPRC) summarise the performance of binary classifiers across threshold values. AUROC is commonly used in ML studies as it provides a single scalar metric for assessing the performance of a model and can be compared between studies. However, the metric does not assess all aspects relevant for evaluating a model and may assign excessive weight to aspects that hold limited relevance for the implementation of the model^20^. Direct comparisons between studies should also be made with caution, as differences in datasets, evaluation protocols, and problem definitions can impact the validity of AUROC-based assessments. The AUPRC is also a common metric but is less used in comparing models across different populations as the baseline is dependent on the underlying distribution of classes in the population.

SHAP values^21^ describe how much each feature contributes to the prediction. For this analysis, SHAP is used to investigate whether models behave differently on a feature-level.

### Software

Python 3^22^ is used for development and analysis. The XGBoost Python Package^23^ is used for model development and SHAP^21^ is used for feature importance.

### Feature selection

The features used are found through a step-wise procedure. First, 41 features are selected as a starting point. These features are mostly the same variables as used in the APACHE IV score and include physiological variables such as *heartrate* and *respiratoryrate*, features describing the admission diagnosis such as the diagnosis category/system, and whether the admission is operative or non-operative. Features are represented by the most extreme measurement, either highest or lowest, relative to a predefined setpoint during the first 24 hours of admission. These setpoints are identical to those used in the Acute Physiology Score III and are already specified in the eICU dataset. This approach captures the most clinically significant deviation from normal values. Categorical variables were transformed using one-hot encoding, whereas numerical variables were retained in their original form without additional transformation. Second, the number of features is reduced from the initial 41 to the 20 deemed most important. The importance is decided by training a model using ten-fold cross-validation and then calculating the importance using SHAP values. These 20 features are the focus of this analysis.

### Complementary feature sets

After the initial feature selection, the 20 features are divided into complementary feature set pairs of size ten. The number ten is chosen as a trade-off between computational cost and sufficient discriminatory performance (AUROC above 0.8 on average). The ten features are found by unordered sampling without replacement. The number of possible combinations is given by the equation

1 $$\begin{aligned} {n \atopwithdelims ()k} = \frac{n!}{k!(n-k)!}, \quad 0 \le k \le n. \end{aligned}$$

Using *n* = 20 and *k* = 10 yields 184 756 possible combinations and 92 378 possible complementary feature set pairs. A selection of 10 000 complementary feature set pairs is chosen as a compromise between exploratory coverage and available computational resources. Since many of the features are correlated, it is not possible to determine the number of pairs required to obtain a full mapping of relationships, therefore 10 000 pairs are selected as a pragmatic balance between scope and feasibility.

The models are developed using an 80/20 train/test split. Training and testing of models using complementary feature sets are done without using cross-validation as this proved too time-consuming. The train/test split is equal for all models so that the results are directly comparable.

### Varying feature set size

The feature set is then further reduced to fewer than ten features to evaluate the impact of varying number of features. New, smaller feature sets are created from four of the feature sets with size ten. The complete set of these subsets is found, and subsets containing fewer than two features are removed. The resulting number of feature subsets is 1013, including the original feature set with size ten. ML models are developed for each feature set using five-fold nested cross-validation to account for variability.

### Single features analysis

Individual features were evaluated by analysing their presence across all 20 000 complementary 10-feature subsets and assessing the association between feature occurrence and model performance. We further examined the 100 feature sets with the highest AUROC and the 100 with the lowest AUROC in greater detail. For every sampled model we recorded the frequency with which each feature appeared, and for the selected high- and low-performing models we computed SHAP values using the TreeExplainer algorithm to quantify feature contributions. The SHAP analyses were used to compare global importance rankings and to inspect explanations within top- and bottom-performing subsets.

## Results

### Initial 41 features

Table 1 shows the overall performance of models developed using all initial 41 features. The APACHE IV mortality prediction is listed as a benchmark. The mean and standard deviation are calculated from ten times ten-fold cross-validation.

**Table 1:** Performance metrics for models with 41 features and APACHE IV.

	All 41 features		APACHE IV	
	Mean	Std	Mean	Std
AUROC	0.855	0.007	0.841	0.010
AUPRC	0.452	0.015	0.421	0.018

Table 2 shows the average feature importance of the top 20 features. The 20 features listed in the table contribute to 87% of the predictions on average while the remaining 21 features contribute to the other 13%. *Age* is considered the most influential feature. Whether the patient was ventilated at the time of the worst respiratory rate (*vent*) is also considered an important factor by the models, as well as the blood urea nitrogen (*bun*) value. The disease-specific, binary variables such as *metastaticcancer*, *hepaticfailure* and *aids* are considered less important by the models.

**Table 2:** Average absolute feature importance using all features.

Feature	Importance
Age	0.097
Vent	0.083
Bun	0.078
Verbal	0.056
Motor	0.054
Heartrate	0.050
Adx	0.044
Respiratoryrate	0.044
Electivesurgery	0.042
Temperature	0.038
Wbc	0.038
Unitadmitsource	0.035
Urine	0.030
Creatinine	0.030
Meanbp	0.028
FiO$_2$	0.026
Hospitaladmitoffset	0.025
Eyes	0.023
Albumin	0.023
Bilirubin	0.022

The number of missing values for the 20 features selected for future analysis is presented in Table A1 in Appendix A. Some patient characteristics are presented in Table B2 in Appendix B.

### Feature sets with ten features

Table 3 shows the overall performance of the 20 000 models with ten features each. The APACHE IV mortality prediction is listed as a benchmark. There is no standard deviation for the APACHE IV since the test set is equal for all models.

**Table 3:** Performance metrics for all 20 000 models with feature set size of ten.

	Ten features				APACHE IV
	mean	std	min	max	
AUROC	0.811	0.008	0.758	0.832	0.839
AUPRC	0.380	0.016	0.273	0.420	0.433

Table 4 shows the overall performance for the 100 models with the highest/lowest AUROC.

**Table 4:** Performance metrics for the 100 best and 100 worst models in terms of AUROC.

	100 highest AUROC				100 lowest AUROC			
	Mean	Std	Min	Max	Mean	Std	Min	Max
AUROC	0.828	0.001	0.826	0.832	0.775	0.006	0.758	0.783
AUPRC	0.396	0.009	0.370	0.413	0.312	0.015	0.274	0.363

**Fig. 2:**
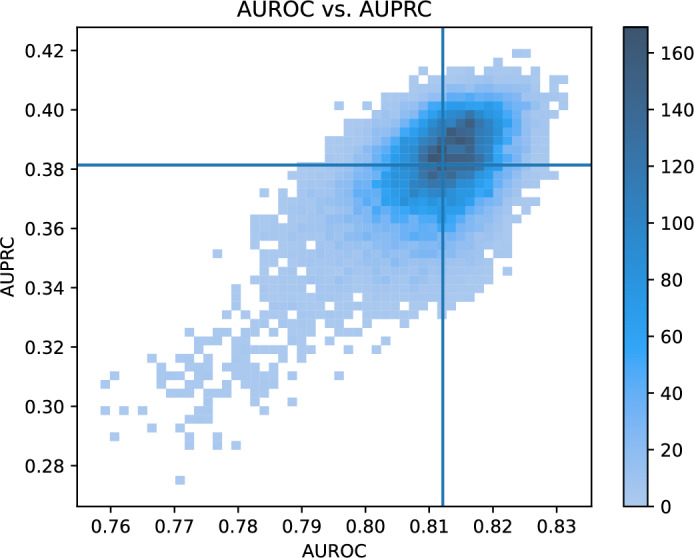
AUROC vs. AUPRC. The lines represent the median AUROC and AUPRC.

Figure 2 shows the AUROC plotted against the AUPRC in a 2D histogram for the 20 000 models. The darker the colour, the higher the number of samples, meaning many models acheived similar performance. The vertical line represents the median AUROC, and the horizontal line represents the median AUPRC. The variance is somewhat higher for values below the median than above, with some results exhibiting significantly worse performance.

**Fig. 3:**
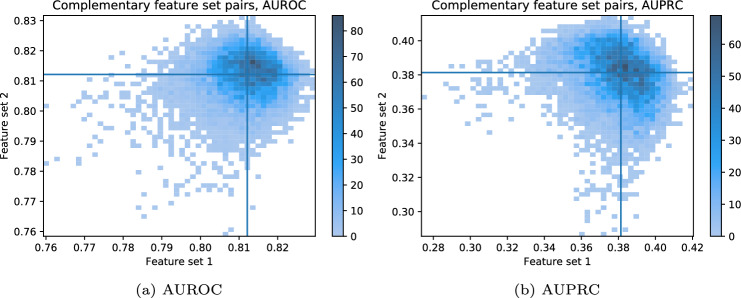
Performance of each complementary feature set pairs plotted against each other. The assignment of ‘Feature set 1’ and ‘Feature set 2’ is arbitrary. The plots change slightly if the the assignment of feature set 1 and 2 is changed.

For each of the 10 000 complementary feature set pairs, the AUROC and AUPRC of feature set 1 and 2 are plotted against each other in the 2D histograms in Fig. 3. Each point in the plots represents a single complementary feature set pair, where the x-axis corresponds to the AUROC/AUPRC of one feature set, and the y-axis corresponds to the AUROC/AUPRC of its complementary set. The designation of which feature set is assigned to the x-axis (labeled ‘Feature set 1’) versus the y-axis (labeled ‘Feature set 2’) is arbitrary and solely reflects the plotting convention. The lines in Fig. 3 represent the median AUROC/AUPRC for all feature sets. The plot shows that most complementary feature set pairs have both feature sets with AUROC between 0.80 and 0.83, with no single feature set demonstrating a clear performance advantage. The same holds for AUPRC values between 0.36 and 0.40.

### Single features analysis

**Fig. 4:**
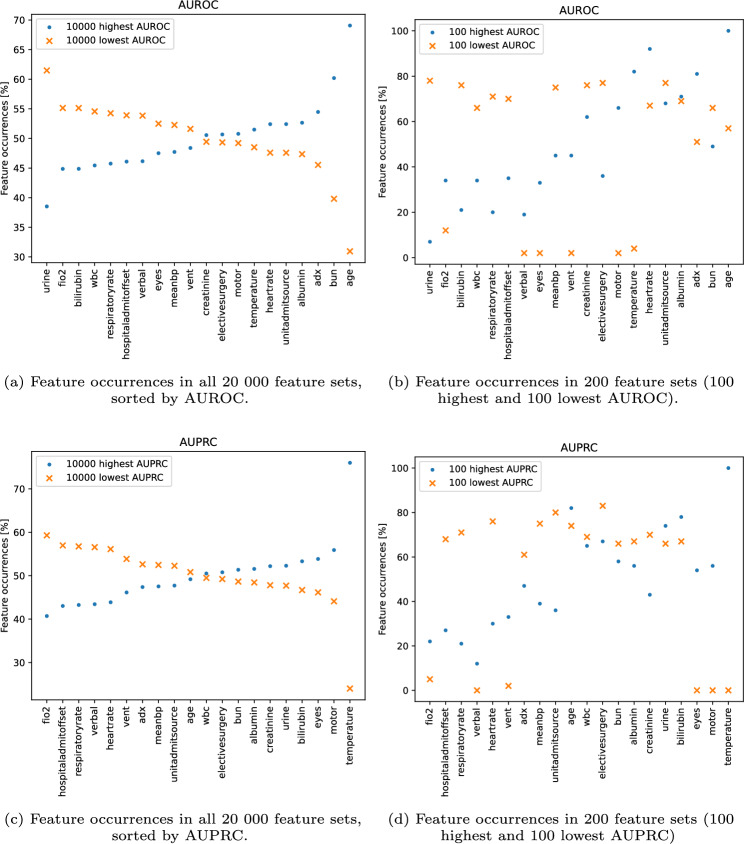
Feature occurrences in all 20 000 feature sets (**a,c**) and feature sets with the 100 highest and 100 lowest AUROC/AUPRC (**b,d**). The x-axes are sorted by highest/lowest feature occurrence in all 20 000 sets.

Figure 4 shows how frequently individual features appear in models with high and low AUROC/AUPRC values. The dots represent the occurrence in feature sets with the highest AUROC/AUPRC, while the crosses represent the occurrence the feature sets with the lowest AUROC/AUPRC. Figure 4a, c show the feature occurrences for the models with the 50% highest and the 50% lowest AUROC/AUPRC. Figure 4b, d show the feature occurrences for models with the 100 highest and 100 lowest AUROC/AUPRC. The figures show no consistent relationship between the frequency of a feature’s occurrence in models with high AUROC and its occurrence in models with high or low AUPRC. *Age* is most frequently present in the feature sets associated with the highest AUROC scores but is not as frequently present in models with high AUPRC. In contrast, *temperature* is more prevalent in the feature sets linked to the highest AUPRC scores but is less dominant in models with high AUROC. *Urine*, appears more frequently in feature sets with low AUROC while slightly more frequently in models with higher AUPRC.

**Fig. 5:**
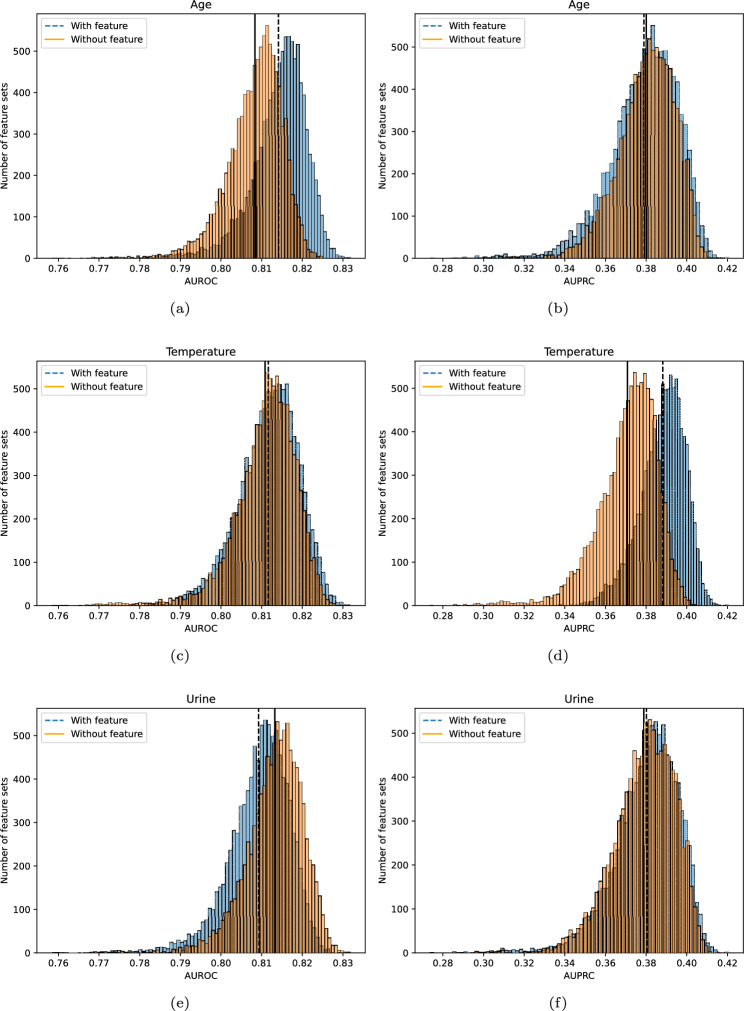
The histograms show the AUROC/AUPRC in the 10 000 feature sets with and 10 000 feature sets without the features *age*, *temperature*, and *urine*. The vertical lines represent the mean AUROC/AUPRC for the feature sets with/without the features.

Figure 5 shows the AUROC/AUPRC for feature sets with and without the features *age*, *temperature* and *urine*. The histograms in Fig. 5a, b show that the feature sets including *age* as a variable have a higher AUROC on average. Feature sets including *temperature* demonstrate slightly better performance in terms of AUPRC while feature sets including *urine* perform worse in terms of AUROC.

### Varying feature set size

**Fig. 6:**
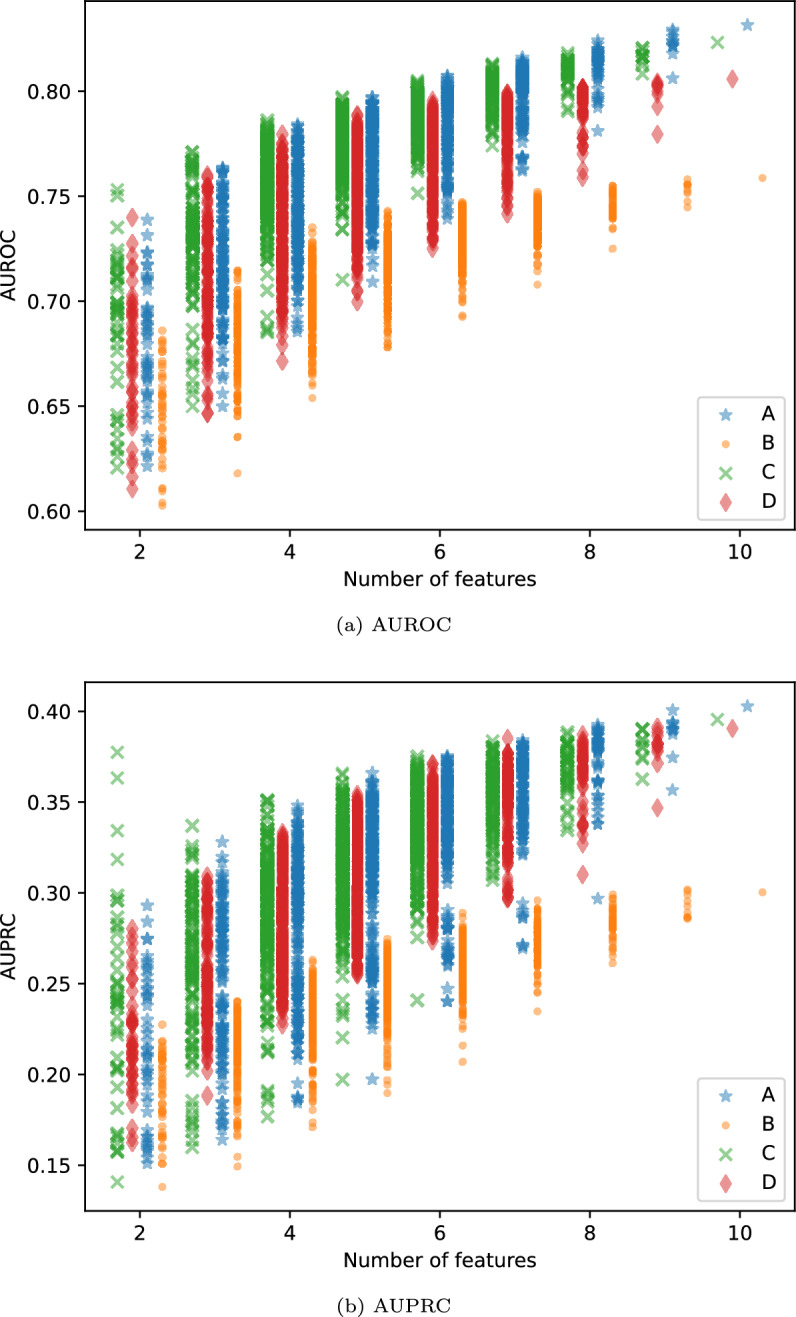
AUROC/AUPRC for feature sets with different sizes. Each point represent a feature sets, one per subset of the feature sets A, B, C, and D listed in Table 5.

Figure 6 shows the AUROC and AUPRC for models with feature set sizes ranging from two to ten, based on the subsets in Table 5. Each colour represents one of the feature sets in the table, and each point represents a subset of the feature set. The total number of feature subsets tested is 1013 per colour. Overall, both AUROC and AUPRC tend to increase as the size of the feature set grows. However, there is significant variation in AUROC/AUPRC across different feature set sizes, and some of the smaller feature sets achieve performance levels comparable to those of larger sets.

**Table 5:** Average AUROC of models using four different feature sets. Set A: Feature set yielding the highest AUROC.Set B: Feature set yielding the lowest AUROC. Set C: Feature 1–10 from Table 2. Set D: Feature 11–20 from Table 2.

Feature set	AUROC (all ten features)	Features
A	0.832	[Adx, age, albumin, creatinine, heartrate, meanbp, motor, respiratoryrate, temperature, unitadmitsource]
B	0.759	[Albumin, bilirubin, electivesurgery, heartrate, hospitaladmitoffset, meanbp respiratoryrate, unitadmitsource, urine, wbc]
C	0.823	[Adx, age, bun, electivesurgery, heartrate, motor, respiratoryrate, temperature, vent, verbal]
D	0.806	[Albumin, bilirubin, creatinine, eyes, fio2, hospitaladmitoffset, meanbp, unitadmitsource, urine, wbc]

### Feature importance

**Fig. 7:**
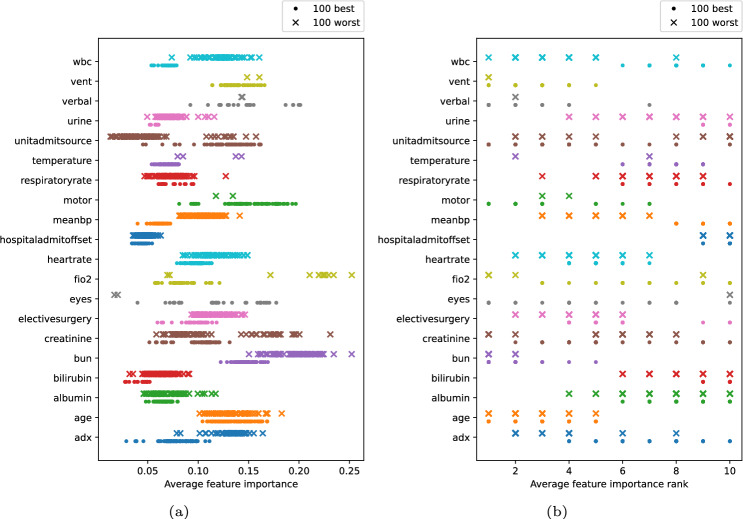
Average feature importance and feature importance rank for the features in the feature sets yielding the 100 best and 100 worst results in terms of AUROC.

Table 4 shows the performance of the 100 best and 100 worst models in terms of AUROC and Fig. 7a shows the average feature importance for the same models. Each dot represents a model. Figure 7b shows the feature importance rank for the same feature sets as in Fig. 7a . The x-axis represents the feature rank. A feature with rank 1 corresponds to the most important feature in a feature set, on average, and rank 10 corresponds to the least important feature.

In Fig. 7a , *age* contributes to 10% to 17% of the predictions on average, regardless of whether the model is among one yielding the highest or lowest AUROC. Other features, such as *FiO2* that contribute to between 6% and 25% and *Creatinine* that contribute to between 6% and 23%, exhibit a broader variance in their contributions to predictions, depending on whether model is among one yielding the highest or lowest AUROC.

## Discussion

### Initial 41 features

From Table 1, we observe that the model created using the initial 41 features performs better than APACHE-IV. Large ML studies often report typical AUROCs in range 0.8–0.9^24^, and our results are within the expected range.

For the initial 41 features, disease-specific, binary variables contribute relatively little to the average model prediction. As most of these conditions are rare, they hold limited statistical power. While the features could be relevant for some individual patient predictions, they are considered less relevant in the population as a whole.

Comparing our selected 20 features with previous ML and prognostic studies shows overlap with physiological and laboratory predictors. In particular, routinely measured vitals and laboratory values are repeatedly reported as relevant predictors of in-hospital and ICU mortality in both classical severity scores (e.g. APACHE and SAPS) and more recent ML models^25–29^. Ventilation status and variables are also common and strong markers of respiratory failure and mortality risks^30^. The Glascow Coma Scale and its components (verbal, motor, eyes) are established predictors of mortality across trauma and medical ICU populations and commonly appear among the top features in predictive models^31^. Age is generally considered a strong predictor across multiple cohorts^27^.

By contrast, variables that reflect administrative context (e.g. *electivesurgery*, *unitadmitsource*, and admission diagnosis (*adx*)) are included variably across studies but have known prognostic relevance. Elective surgical admissions generally carry lower ICU mortality than emergency/non-elective admissions, and pre-ICU admission site (ED, ward, operating room, transfer), and primary admission diagnosis add important information about case mix and baseline risk^32–34^. These features may therefore improve model performance even if they are used less consistently than physiological and laboratory measurements.

Overall, the overlap between our top-20 features (Table 2) and prior work indicates these predictors reflect established, clinically plausible risk factors rather than spurious model artefacts. Variation in feature ranking across studies are expected as differences in cohorts, outcome definitions, variable selection and coding, and choice of algorithm and metrics all influence importance estimates.

We also emphasise three different caveats that affect the interpretation of feature importances. First, the Table 2 reports average importances across folds and patients, and not the relevant features for individual patient predictions. A feature with low average can still be highly relevant for a particular patient prediction. Second, feature importances describes the model not the real world. A feature highly relevant for a model prediction is not equivalent to the feature being highly relevant for the actual outcome, nor does it mean that the feature would hold the same relevance in a different model. Third, missing values can inherently carry information, their prevalence may influence the results of the analysis. The underlying causes of the missing values presented in Suppl. Table A1 in Appendix A are unknown but are likely caused by multiple factors. Future research should explore this further to gain a deeper understanding of its impact.

### Feature sets with ten features

The performance of models using ten features is lower than the APACHE IV performance, as seen in Table 3. The performance is also lower than models using the initial 41 features in Table 1. These results are as expected as the 41 features contain more information than ten features, and the number of features is not so high that the models experience the challenges associated with high dimensionality.

Comparing the results to other studies is not straightforward as similar studies use different datasets, populations, types of features, preprocessing techniques, algorithms, performance metrics, and criteria. Compared to other mortality prediction models the mean AUROC when using ten features is in the lower range^24^. Still, AUROC is not sufficient as the sole metric for evaluating or deciding between models. The variation in the proportion of deceased patients in different populations makes it more challenging to compare AUPRC with other studies.

Figure 2 shows a positive relationship between AUROC and AUPRC. The majority of the models achieve an AUROC between 0.80 and 0.83, and an AURPC between 0.36 and 0.40. These results demonstrate that multiple combinations of features yield similar performance. As the features are not independent, and the 10 000 feature set pairs do not cover all possible combinations, we cannot exclude the possibility that some remaining combinations would exhibit different behaviour. Nevertheless, the results are consistent with the Rashomon effect stating that for a given dataset, there may exist multiple solutions with comparable performance^35^.

Figure 3 indicates no clear relationships between complementary feature set pairs regarding the AUROC and AUPRC. Most complementary feature set pairs have both values in a specific range, with some outliers. The results show that having a feature set with a high AUROC or AUPRC does not implicate a low performance of the complementary feature set. For some outliers, both feature sets have similar performance. The lack of a clear trend indicates that different feature sets can have comparable performance.

### Single features analysis

As seen in Fig. 4a, b , *age* occurs in the majority of feature sets with the highest AUROC. This frequent occurrence could indicate that including *age* in the feature set is beneficial. However, Fig. 4c shows that *age* occurs in only half of the 10 000 feature sets with the highest AUPRC. The *temperature* and *urine* occurrences are also not consistent between AUROC and AUPRC. The feature sets common for the 10 000 models with the highest AUROC and 10,000 models with the highest AUPRC are found in the top right corner in Fig. 2. The histograms in Fig. 5 indicate that having a specific feature in the feature set does not guarantee better overall performance.

These results indicate that the feature selection process should consider the distinct trade-offs associated with different performance metrics. While AUROC reflects a model’s ability to discriminate between classes, emphasising sensitivity and specificity, the AUPRC focus on the precision-recall trade-off. Consequently, a feature that significantly contributes to the overall discrimination might not contribute equally well to high precision and recall, or other types of performance metrics.

### Varying feature set size

As expected, the AUROC tends to increase as the number of features grows, particularly given the initially small feature set size and given that all added features are considered relevant to the outcome. While the general trend within each feature subset shows that AUROC improves with more features, some smaller feature sets containing only three or more features outperform certain ten-feature models. For instance, the feature set [*motor*, *heartrate*, *creatinine*] with an AUROC of 0.763 surpasses Subset B in Table 5 with an AUROC of 0.759. Increasing the number of features above ten further increases the AUROC, though investigating the optimal number of features falls outside the scope of this paper.

Despite being based on the ten features with the lowest feature importance, Subset D performs more similar to the best performing subset (Subset A) and the subset based on the features with the highest feature importance (Subset C) than the subset with the lowest AUROC (Subset B). While some features are deemed less important when considering the full feature set of 41 features, they may provide complementary information when combined differently. Additionally, individual feature importance may be underestimated when interactions between features are not fully considered. The results suggest that even features with lower average importance may hold predictive value in certain contexts, supporting the broader finding that multiple feature combinations can yield similarly strong results.

### Feature importance

Figure 7a, b should be analysed with the help of Fig. 4b . A small variance in importance could indicate that a feature is consistently one of the most or least important features across different models and may imply that the feature has a stable influence on the model’s performance, regardless of the specific subset of features being used. However, low feature occurrence in the selected feature sets could also lead to small variance in importance rankings. Features that rarely appear in the feature sets yielding the best or worst overall results, such as *eyes* and *vent*, may show little variation in importance simply because the low occurrence limits proper assessment of their impact.

While *age* is considered the most important feature when using all 41 features (Table 1), *age* is not always considered the most important feature as seen in Fig. 7b . Here, *age* contributes more to the predictions on average and is considered between the most important and fifth most important feature when looking at feature sets with 10 features. *Age* is not considered one of the least important features, regardless of the feature combination. The importance of features like *eyes* and *creatinine* depends more on the feature combination than *age* as the feature importance rank has a larger variance. In these cases, the infrequent inclusion of a feature might prevent it from being recognised as important, even though it could potentially have value in certain contexts or combinations. Therefore, the interplay between feature importance and occurrence needs to be carefully interpreted, as low occurrence does not necessarily indicate irrelevance, but rather may reflect the feature’s limited participation in subsets associated with higher performance.

High feature correlation can lead to uncertainty in determining the feature importance as it becomes more challenging to distinguish which of the features are contributing to the prediction. Highly correlated features can inflate the importance of one of the features while diminishing the importance of the other. This potential bias should be further investigated to ensure a more reliable interpretation of feature importance and its impact on model performance.

From a clinical perspective, our findings underline that predictive models for ICU mortality must be evaluated not only by their performance metrics but also by their clinical interpretability. In daily ICU practice, decisions are rarely based on a single variable but rather on the integration of multiple, sometimes redundant cues. The observation that different feature sets can achieve comparable discrimination reflects this reality and highlights the need for models that support—rather than replace—clinicians’ judgement by providing transparent, context-relevant predictions.

### Limitations and future work

Both training and testing were conducted on a single dataset, so the generalisability of the results to other hospitals, measurement practices, and time periods remains untested. Moreover, both patient- and feature-selection may have introduced bias in the dataset. By excluding patients with short or multiple stays and discarding records with missing values and limits the results applicability to broader populations. Future work should focus on external validation and expanding to other datasets such as MIMIC III and IV. Robustness and consistency should be assessed across algorithms, preprocessing techniques, and evaluation metrics.

We also recommend expanding the number of complementary feature-set pairs in combination with different feature importance calculations and extend the interpretability analysis to the individual patient level. To better understand these results, feature values and importance should be studied together. Without knowing the feature values, we cannot determine how they influence predictions. For example, while *age* might be a significant predictor of mortality, we need to discern whether it is a better predictor for older or younger individuals. Furthermore, we need to understand the direction of each feature’s influence. Does old age increase the likelihood of predicting mortality, or does it suggest a greater chance of survival? Similarly, other features might have complex interactions that only become clear when values and importance are considered together. By integrating both aspects, we can gain deeper insights into the predictive power and behaviour of each feature within the models. Other performance metrics should also be included as AUROC and AUPRC do not sufficiently describe the performance of a model. In particular, individual-level case studies can provide valuable insights into how features interact in specific patient scenarios, highlighting nuances that are less apparent from aggregate numbers. Future research could incorporate such individual-level analyses as a natural extension of this work, helping to link model explanations with clinically meaningful patterns.

For future studies, it would also be interesting to consider more than ten features per model as well as investigate the specific feature combinations. Including multiple highly correlated features in the same feature set limits the potential of more diverse information if the feature set size is limited to ten. Additionally, testing different feature importance techniques could help assess the robustness of feature rankings. Investigating whether certain feature sets can be identified as likely to yield good or poor performance before training could also be an interesting direction for further research.

Translating the results into clinical practice demands additional work and development. The findings of this study are general and cannot be directly applied in clinical decision-making. Standard metrics such as AUROC and AUPRC reflect overall discrimination rather than clinical utility at relevant operating thresholds, and future work should examine task-specific metrics that align with the specific clinical setting^36^. Models intended for implementation require different design choices than those for exploratory research, including consideration of outcome-action pairing, prediction timing, and presentation of results^37,38^. Providing clinicians with clear, task-relevant explanations of the features driving a prediction may support informed decisions, but these explanations describe model behaviour rather than real-world effects. Different models can yield different explanations for the same patients, highlighting the need for cautious interpretation. Explanations such as features relevant to the prediction should therefore be integrated into workflows in ways that deliver actionable and verifiable information, such as confidence estimates or indicators of model unreliability. Importantly, models should be designed to support rather than replace clinical judgement, and their use requires context-sensitive implementation and governance to ensure patient safety and clinical value^39^.

## Conclusion

This study highlights some of the complexities of feature selection and feature sets in machine learning, particularly in the context of predicting in-hospital mortality in the ICU. The study’s findings reveal insights into the predictive capabilities of models using different feature sets, reflecting the Rashomon effect where different models achieve comparable performance. This holds true for both overlapping and complementary feature combinations.

The results indicate the significance of feature combinations over individual features alone. Features are weighted differently depending on the feature combination, both in terms of percentage and rank. The results also show that feature selection should be evaluated in combination with performance metrics as different features may impact performance differently dependent on the chosen evaluation criteria.

In summary, identifying which and how specific features influence mortality is challenging. Effective feature selection requires considering both individual features and their interactions within feature combinations, as these can significantly impact model behaviour. It is important to recognise that feature importances cannot be straightforwardly applied to real-life clinical situations. Even with sound explanations, features describe how a model behaviour, and not real-world causal effects. In addition, as the results show, feature importances depends on the complex interplay of features and varies between different models. Thus, while the study provides valuable insights into predictive modelling, it emphasises the need for cautious and nuanced interpretation and application of model outcomes in clinical practice.

## Supplementary Information

Supplementary Information.

## Data Availability

The dataset used is from the publicly available eICU Collaborative Research Database v2.0 (https://eicu-crd.mit.edu/).

## Abbreviations

APACHE: Acute Physiology and Chronic Health Evaluation
AUPRC: Area under the precision recall curve
AUROC: Area under the receiver operating characteristics curve
FN: False negative
FP: False positive
ICU: Intensive care unit
ML: Machine learning
SHAP: SHapley Additive exPlanations
TN: True negative
TP: True positive
XGBoost: eXtreme Gradient Boosting

## Code availability

The code for reproducing the work in this study will be published on GitHub (https://github.com/Explainable-Hospital-Mortality/impact-of-feature-combinations).
