# Supplementary material for: The impact of feature combinations on machine learning models for in-hospital mortality prediction
Source: Sci Rep. 2025 Nov 7;15:39119. doi: 10.1038/s41598-025-26611-y (PMC12594765; doi:10.1038/s41598-025-26611-y)
Supplement: Supplementary file 1 — Supplementary Information. [file 41598_2025_26611_MOESM1_ESM.pdf]

Appendix A Missing values

Variable	Total (n=73 210)	Deceased (n=7257)	Alive (n=65 953)
age	0	-	-
vent	0	-	-
bun	12469 (17.0%)	857 (11.8%)	11612 (17.6%)
verbal	964 (1.3%)	192 (2.6%)	772 (1.2%)
motor	964 (1.3%)	192 (2.6%)	772 (1.2%)
heartrate	0	-	-
admission diagnosis	0	-	-
respiratoryrate	0	-	-
electivesurgery	56996 (77.9%)	6537 (90.1%)	50459 (76.5%)
temperature	2546 (3.5%)	268 (3.7%)	2278 (3.5%)
wbc	14543 (19.9%)	1139 (15.7%)	13404 (20.3%)
unit admit source	0	-	-
urine	33224 (45.4%)	3470 (47.8%)	29754 (45.1%)
creatinine	12140 (16.6%)	853 (11.8%)	11287 (17.1%)
meanbp	0	-	-
fio2	52977 (72.4%)	3627 (50.0%)	49350 (74.8%)
hospitaladmitoffset	870 (1.2%)	73 (1.0%)	797 (1.2%)
eyes	964 (1.3%)	192 (2.6%)	772 (1.2%)
albumin	42387 (57.9%)	3232 (44.5%)	39155 (59.4%)
bilirubin	45000 (61.5%)	3514 (48.4%)	41486 (62.9%)

Table A1: Number of missing values for the 20 variables used for further analysis.

Appendix B Patient characteristics

Variable	Total	Deceased	Alive
Age (yrs), median (IQR)	66 (54 - 77)	71 (60 - 81)	65 (53 - 76)
Albumin (g/dL), median (IQR)	2.9 (2.4 - 3.4)	2.5 (2.0 - 3.0)	2.9 (2.5 - 3.4)
Bilirubin (mg/dL), median (IQR)	0.7 (0.4 - 1.1)	0.9 (0.5 - 1.7)	0.7 (0.4 - 1.1)
BUN[a] (mg/dL), median (IQR)	19 (13 - 32)	30 (19 - 48)	18 (13 - 30)
Creatinine (mg/dL), median (IQR)	0.99 (0.72 - 1.58)	1.49 (0.90 - 2.47)	0.94 (0.71 - 1.48)
Electivesurgery, n (%)	14324 (88.3%)	527 (73.2%)	13797 (89.0%)
Eyes[b], median (IQR)	4 (3 - 4)	3 (1 - 4)	4 (3 - 4)
FiO2 (%), median (IQR)	50 (40 - 80)	60 (50 - 100)	50 (40 - 80)
Heartrate (bpm), median (IQR)	105 (89 - 121)	114 (97 - 132)	104 (88 - 120)
Hospital admission offset (min), median (IQR)	-240 (-652 - -76)	-226 (-1094 - -69)	-242 (-633 - -77)
Males, n (%)	40085 (54.8%)	3971 (54.7%)	36114 (54.8%)
Mean blood pressure (mmHg), median (IQR)	65 (53 - 126)	58 (47 - 129)	66 (54 - 126)
Motor[b], median (IQR)	6 (6 - 6)	5 (3 - 6)	6 (6 - 6)
Respiratory rate (breaths/min), median (IQR)	28 (11 - 36)	31 (14 - 39)	28 (11 - 36)
Temperature (^{o}C), median (IQR)	36.4 (36.1 - 36.7)	36.3 (35.6 - 36.7)	36.5 (36.2 - 36.7)
Urine (mL/day)	1446 (804 - 2389)	955 (424 - 1849)	1494 (853 - 2437)
Ventilated, n (%)	20086 (27.4%)	3953 (54.5%)	16133 (24.5%)
Verbal[c], median (IQR)	5 (3 - 5)	3 (1 - 5)	5 (4 - 5)
WBC[d] (1000/uL), median (IQR)	10.7 (7.6 - 15.7)	13.7 (8.3 - 19.7)	10.5 (7.6 - 15.3)
**Admission diagnosis, n (%)**			
Cardiovascular	33133 (45.3%)	3775 (52.0%)	29358 (44.5%)
Neurologic	13311 (18.2%)	1031 (14.2%)	12280 (18.6%)
Respiratory	9841 (13.4%)	1286 (17.7%)	8555 (13.0%)
Gastrointestinal	7528 (10.3%)	623 (8.6%)	6905 (10.5%)
Trauma	3619 (4.9%)	275 (3.8%)	3344 (5.1%)
Metabolic/Endocrine	2568 (3.5%)	70 (1.0%)	2498 (3.8%)
Genitourinary	1811 (2.5%)	110 (1.5%)	1701 (2.6%)
Musculoskeletal/Skin	810 (1.1%)	37 (0.5%)	773 (1.2%)
Hematology	483 (0.7%)	49 (0.7%)	434 (0.7%)
Transplant	106 (0.1%)	1 (0.0%)	105 (0.2%)
**Admit source, n (%)**			
Emergency Department	36973 (50.5%)	3761 (51.8%)	33212 (50.4%)
Operating Room	12330 (16.8%)	531 (7.3%)	11799 (17.9%)
Floor	9233 (12.6%)	1393 (19.2%)	7840 (11.9%)
Direct Admit	5992 (8.2%)	686 (9.5%)	5306 (8.0%)
Recovery Room	3120 (4.3%)	133 (1.8%)	2987 (4.5%)
Other Hospital	2114 (2.9%)	315 (4.3%)	1799 (2.7%)
Acute Care/Floor	1364 (1.9%)	168 (2.3%)	1196 (1.8%)
Step-Down Unit (SDU)	1295 (1.8%)	236 (3.3%)	1059 (1.6%)
PACU	593 (0.8%)	28 (0.4%)	565 (0.9%)
Chest Pain Center	173 (0.2%)	6 (0.1%)	167 (0.3%)
ICU to SDU	14 (0.0%)	0 (0.0%)	14 (0.0%)
Observation	5 (0.0%)	0 (0.0%)	5 (0.0%)
Other	4 (0.0%)	0 (0.0%)	4 (0.0%)

Table B2: Patient characteristics. For binary variables, the percentages are calculated from the non-missing values.

[a]Blood urea nitrogen [b]Part of Glasgow Coma Scale [c]White blood cells
